# Adductor canal block combined with local infiltration analgesia with morphine and betamethasone show superior analgesic effect than local infiltration analgesia alone for total knee arthroplasty: a prospective randomized controlled trial

**DOI:** 10.1186/s12891-022-05388-5

**Published:** 2022-05-19

**Authors:** Zhen-Yu Luo, Qiu-Ping Yu, Wei-Nan Zeng, Qiang Xiao, Xi Chen, Hao-Yang Wang, Zongke Zhou

**Affiliations:** 1 Department of Orthopedics, West China Hospital/West China School of Medicine, Sichuan University, 37# Wuhou Guoxue road, Chengdu, 610041 China; 2 Orthopedic Research Institute, West China Hospital, Sichuan University, Chengdu, 610041 China; 3 Health Management Center, West China Hospital/West China School of Medicine, Sichuan University, Chengdu, 610041 China; 4 Department of Operative Dentistry and Endodontics, West China Hospital of Stomatology, Sichuan University, Chengdu, 610041 China

**Keywords:** Local infiltration analgesia, Adductor canal block, Morphine, Betamethasone

## Abstract

**Background:**

Local infiltration analgesia (LIA) and adductor canal block (ACB) provide postoperative analgesia for total knee arthroplasty (TKA). ACB blocks the saphenous nerve and has smaller impacts on quadriceps muscle weakness. ACB theoretically does not have enough analgesic effects on posterior sensory nerves. LIA may increase its analgesic effects on the posterolateral knee. The purpose of this study was to evaluate whether ACB combined with a LIA cocktail of ropivacaine, morphine, and betamethasone has superior analgesic effect than LIA for TKA.

**Methods:**

A total of 86 patients were assessed for eligibility from February 2019 to May 2019. 26 of those were excluded, and 60 patients were divided into 2 groups by computer-generated random number. Group A (LIA group) received LIA cocktail of ropivacaine, morphine and betamethasone. Group B (LIA+ ACB group) received ultrasound-guided ACB and LIA cocktail of ropivacaine, morphine and betamethasone. Postoperative visual analogue scale (VAS) resting or active pain scores, opioid consumption, range of motion (ROM), functional tests, complications and satisfaction rates were measured. The longest follow-up was 2 years.

**Results:**

Two groups have no differences in terms of characteristics, preoperative pain or function (*P* > 0.05). ACB combined with LIA had significantly lower resting and active VAS pain scores, better ROM, better sleeping quality and higher satisfaction rates than LIA alone within 72 h postoperatively (*P* < 0.05). Complications, or adverse events and HSS score, SF-12 score were observed no significant differences within 2 years postoperatively.

**Conclusions:**

Adductor canal block combined with Local infiltration analgesia provide better early pain control. Although the small statistical benefit may not result in minimal clinically important difference, Adductor canal block combined with Local infiltration analgesia also reduce opioid requirements, improve sleeping quality, and do not increase the complication rate. Therefore, Adductor canal block combined with Local infiltration analgesia still have good application prospects as an effective pain management for total knee arthroplasty.

**Trial registration:**

Chinese Clinical Trial Registry, ChiCTR1900021385, 18/02/2019.

## Introduction

The proportion of total knee arthroplasty (TKA) procedures judged as “excellent” quality has reached more than 90% [1]; however, the patient satisfaction rate of TKA is less than 80%, and patients still experience perioperative pain [2]. Enhanced recovery after surgery (ERAS) protocols for TKA have produced significant clinical and economic benefits, and pain management is an important part of these protocols for early recovery and patient satisfaction [3]. ERAS promotes multimodal analgesia as the essential preventive strategy to ensure postoperative pain control. Multimodal analgesia is a multidisciplinary approach to pain management, taking advantages of the additive or synergistic effects of various analgesic methods to maximize the analgesic effects and minimize the complications caused by these drugs [4].

Local infiltration analgesia (LIA) is given as a peri- and intra-articular injection during the operation and has been reported to be a safe and effective method, which not only reduces postoperative pain, but also significantly decreases the consumption of opioids [5–7]. The most commonly utilized LIA formulas consist of a single local anesthetic such as ropivacaine or bupivacaine. However, a recent technique known as the cocktail therapy used a combination of injected local anesthetics, with the addition of steroids, epinephrine, NSAID, and opioids, to enhance the analgesic effect [4]. LIA formulas that include opioids such as morphine have been shown to be effective for pain relief. Betamethasone is a long-acting glucocorticoid with potential anti-inflammatory and pain-controlling effect [2, 6, 8, 9]. However, the safety of these combinations lacks long-term follow-up.

Adductor canal block (ACB) is a new technique that blocks the saphenous nerve and vastus medialis nerve under ultrasound guidance to achieve an analgesic effect. ACB has a similar analgesic effect to the previously common femoral nerve block (FNB) procedure. However, with the possibility of motor block, FNB will lead to instability of functional exercise after TKA, increasing the risk of falling. In addition, FNB did not block the obturator nerve and related branches of sciatic nerve. ACB resolves the defect that the injection of drugs into the femoral triangle does not diffuse the socket, greatly increases the success rate of analgesia, minimizes the strength of the affected limb, and maintains the stability of the patient’s early functional exercises. ACB has smaller impacts on quadriceps muscle weakness which translates to a lower risk of postoperative falls [10–12]. However, first, a relatively short duration of analgesia effects limited the clinical application. Second, ACB theoretically blocks only the nerves innervating anterior parts of the knee joint and does not have enough analgesic effects on the posterior or lateral sensory nerves of the knee. Procedures during the operation, such as posterior capsule or ligament release, may cause severe pain on the posterior and lateral sides of the knee joint [13]. LIA can be injected into the peri- and intra-articular regions, and may increase its analgesic effects on the posterolateral knee and lengthen its period of effectiveness. Thus, combined ACB and LIA may improve and prolong analgesia. However, it remains controversial whether ACB combined with LIA provides superior analgesia compared with LIA alone [13–16]. Moreover, the follow-up time for analgesic effect and complications in these studies is relatively short.

The objective of this randomized controlled trial was to evaluate the analgesic effect of ACB combined with a LIA cocktail of ropivacaine, morphine, and betamethasone for TKA. The hypothesis was that ACB combined with LIA would provide similar or better postoperative analgesic effects and pain control than LIA alone. The follow-up time was 2 years. This study will be of great significance for clinicians and researchers to select appropriate analgesic models for perioperative pain management of TKA.

## Materials and methods

This prospective single-center randomized controlled trial was approved by the Institutional Review Board of Sichuan University, West China Medical Center, China. The work was registered in the Chinese Clinical Trial Registry (Register date: 18/02/2019, ID number: ChiCTR1900021385). Informed consent was obtained from all participants.

### Patients and exclusion criteria

Patients were enrolled undergoing unilateral primary TKA between February 2019 and May 2019 in the trial. Patients were included with diagnoses of primary osteoarthritis or rheumatoid arthritis who were undergoing unilateral primary TKA and provided written informed consent. The included patients were aged from 18 to 80 years old, gender and other demographic data were not limited. Patients were excluded who declined to participate; those who had not been diagnosed with primary osteoarthritis or rheumatoid arthritis, for example, traumatic arthritis; those who had an active local or systemic infection, any contraindication for general anesthesia, or an American Society of Anesthesiologists (ASA) physical health classification above grade III; those who were allergic or intolerant to local analgesia, NSAIDs or opioids; those who had progressive neurological deficits in the femoral or sciatic nerve distribution; those who had undergone another surgery in the past 6 months; those who had utilized opioids or neurological pain medicine within the past 6 months; those who had preoperative hepatic or renal dysfunction; those who had severe cardiac comorbidities or coagulopathy; and those who were pregnant at the time of the study.

### Randomization procedure

**Fig. 1:**
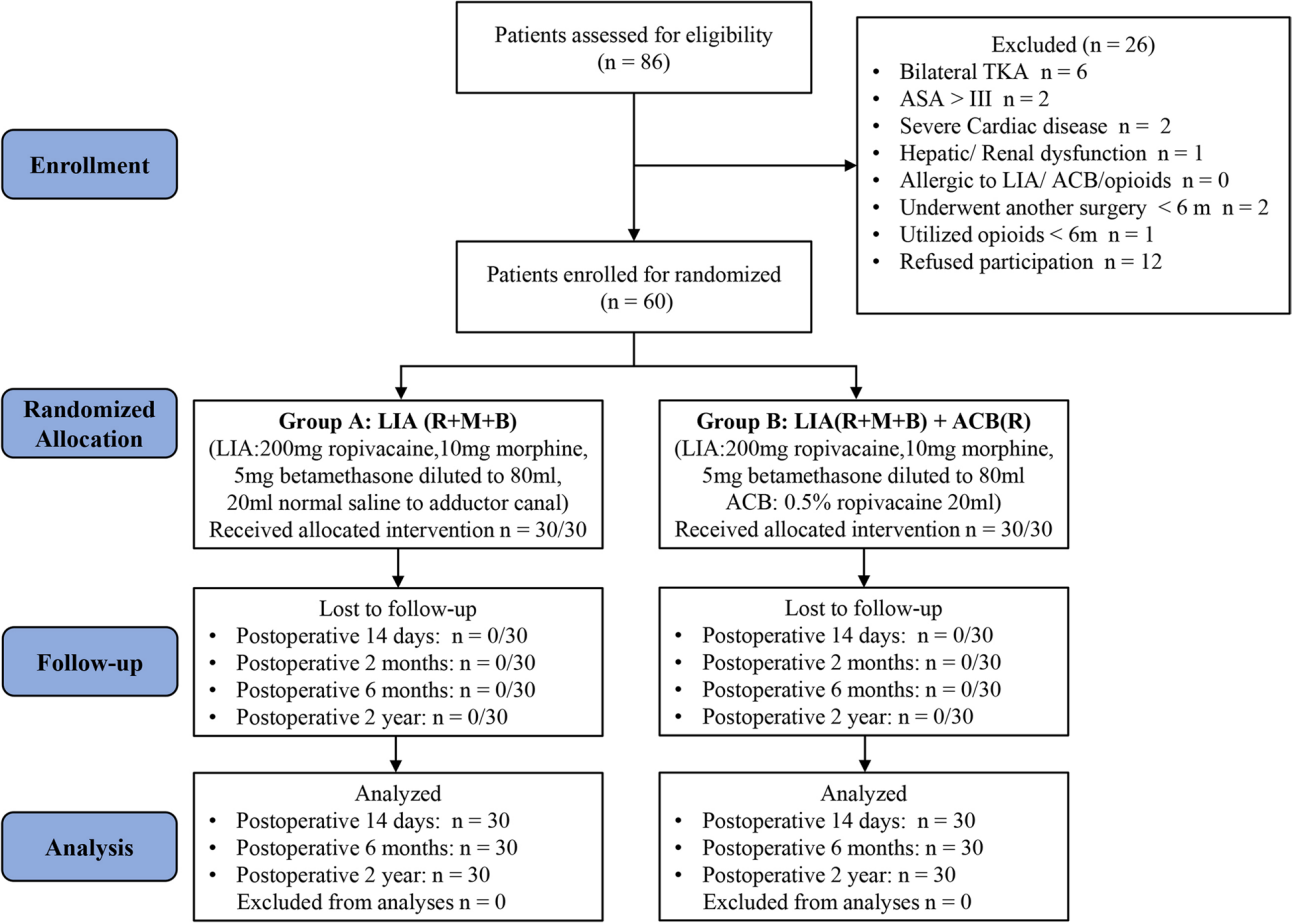
Flow chart of the study procedure

Patients were randomized into 2 groups using a computer-generated list of random numbers. If the random number is odd, they are divided into Group A, and if the random number is even, they are divided into Group B. The allocation of random numbers was performed by other nurses other than surgeons and follow-up researchers. Patients and researchers did not know the grouping status until the data analysis stage at the end of the study. Group A (LIA group, 30 patients) received LIA with 200 mg ropivacaine, 10 mg morphine, 5 mg betamethasone diluted with normal saline to 60 ml. Group B (LIA + ACB group, 30 patients) received ultrasound-guided ACB with 0.5% ropivacaine 20 ml (100 mg) and LIA with 200 mg ropivacaine, 10 mg morphine, 5 mg betamethasone diluted with normal saline to 60 ml. Group A also received 20 ml normal saline injection into the adductor canal to keep the total amount of fluid injected equal for blinding. The CONSORT flow diagram is shown in Fig. 1.

### Anesthesia intervention, surgical procedure and perioperative management

All patients received general anesthesia. ACBs were performed on patients after general anesthesia but before skin incision. ACBs were performed using real-time ultrasonography with a color Doppler ultrasound machine. A high-frequency linear array ultrasonic transducer was used to scan the middle of the thigh to identify the adductor canal. The anterolateral hyperechoic structures, such as the saphenous nerve, were identified as the injection target, and 0.5% ropivacaine or normal saline 20 ml was injected. Local periarticular infiltration analgesia was injected into patients in all groups after osteotomy was completed but before the prostheses were implanted. All Groups received 200 mg ropivacaine, 10 mg morphine, 5 mg betamethasone diluted with normal saline to 60 ml. LIA was performed around the medial collateral ligament, lateral collateral ligament, medial capsule, lateral capsule, posterior capsule, vastus medialis obliquus muscle and quadriceps tendon, prepatellar tissues, and subcutaneous tissues. No tourniquet was used during the operation.

All patients were given second generation cephalosporin to prevent infection. Additionally, all patients underwent postoperative multimodal pain management according to the standard practice at the study institution. Parecoxib was administered at 20 mg twice a day to every patient from immediately after the operation until discharge, and oxycodone or morphine was used when patients reported an immediate pain which was greater than 4 on a 0–10 visual analogue scale (VAS). Continuous movement exercises were started postoperatively to promote recovery.

### Outcome measures

Preoperatively, demographic and baseline characteristics were collected, including preoperative (VAS) pain scores, Hospital for Special Surgery (HSS) scores, range of motion (ROM), as listed in Table 1.

**Table 1:** Baseline of characteristics and perioperative demographics

	Group A (*n* = 30)	Group B (*n* = 30)	t / χ^2^	P
Age	65.3 ± 4.86	65.4 ± 5.22	0.076	0.9391^a^
Gender (Famle/Male)	22/8	23/7	0.089	0.7656^b^
BMI (kg/m^2^)	24.93 ± 5.24	24.83 ± 4.67	0.078	0.9381^a^
Diagnoses n (%)			0.162	0.6876^b^
Osteoarthritis	27 (90%)	26 (86.6%)		
Rheumatoid arthritis	3 (10%)	4 (13.3%)		
Side (L/R)	16/14	15/15	0.067	0.7961^b^
Comorbidities				
Hypertension	11 (36.7%)	13 (43.3%)	0.278	0.5982^b^
Diabetes	5 (16.6%)	4 (13.3%)	–	1.000^c^
COPD	2 (6.6%)	2 (6.6%)	–	1.000^c^
Hypothyroidism	0 (0%)	1 (3.3%)	–	1.000^c^
Preoperative VAS				
Rest	2.6 ± 0.8	2.6 ± 1.0	0.000	1.0000^a^
Activity	4.6 ± 1.5	4.5 ± 1.2	0.285	0.7766^a^
Preoperative ROM	104.3 ± 10.8	103.3 ± 10.7	0.360	0.7200^a^
Preoperative HSS	45.3 ± 6.2	48.8 ± 9.4	1.702	0.0940^a^
ASA (I/II/III)	0/25/5	0/24/6	–	1.0000^c^

*BMI* body mass index, *COPD* chronic obstruct pulmonary disease, *ASA* American Society of Anesthesiologists

^a^ The P value represented the result of the Student’s t test for continuous variables between 2 groups

^b^ The *P* value represented the result of the Pearson’s χ^2^ test for discontinues variables between 2 groups

^c^ The P value represented the result of the Fisher’s exact test for discontinues variables between 2 groups

*P* < 0.05 indicated significant differences

The primary outcomes were pain evaluation and functional recovery. Pain at rest (resting pain) and pain during physical activity (active pain) were assessed on a VAS, in which patients assigned their pain a numeric rating score (NRS) ranging from 0 to 10 points (0 = no pain, 10 = worst imaginable pain). Based on these s cores, the intensity of pain was sorted into five levels: very painful (8–10), painful (6–8), moderately painful (4–6), slightly painful (2–4), and nearly painless (0–2). Pain evaluation were collected at 6 hours postoperatively (PO 6 h) as well as PO 12 h, 24 h, 36 h, 48 h, 72 h, 7 days (7d), 14d, 2 months (2 m), 6 m,1 year (1y) and 2y. The location of pain was evaluated at PO 48 h. Opioid consumption was measured from the completion of surgery to PO 48 h and 72 h. Total opioid consumption was calculated by converting opioids consumed to morphine equivalents (ME). All patients had similar pain management from discharge to removal of stitches (approximately PO 14 d); therefore, analgesics utilized from discharge to PO 14 d were not recorded. Any other analgesics utilized from PO 14 d to PO 2 m were recorded. Knee ROM was measured at PO 24 h, 48 h, 72 h, 7d, 14d, 2 m, 6 m, 1y and 2y. The straight leg raise test was conducted and recorded within PO 72 h, evaluating whether and when patients could complete the action without using their hands. Similarly, we recorded whether patients could get out of bed and try to stand with the help of a walking aid, then, walking 20 m with a walking aid was also recorded. Sleeping quality was evaluated by Epworth sleepless scale (ESS), which used self-administered and validated questionnaires to measure patients’ daytime drowsiness, and scores were used to quantify excessive daytime drowsiness (somnolence more than 6 points, excessive drowsiness more than 11 points, dangerous drowsiness points more than 16 points). ESS scores were recorded on the morning of day 1, day 2 and day 3.

The secondary outcomes included adverse events, length of hospitalization, satisfaction rate, HSS score and SF-12 score. Adverse events occurring within PO 2 m were recorded. Postoperative nausea (PON) was defined as the unpleasant sensation associated with awareness of the urge to vomit. Postoperative vomiting (POV) was defined as the forceful expulsion of gastric contents from the mouth. The presence or absence of PON/V was recorded, and the severity level was assessed on a 4-point scale (0 = none, 1 = light, 2 = moderate, 3 = severe). Dizziness, hypotension, uroschesis, and pruritus, wound infection were also recorded. The length of a patient’s postoperative hospital stay was defined as the time from the completion of surgery to discharge. At PO 72 h, we evaluated the patients’ satisfaction with their functional recovery and pain control to calculate the satisfaction rates. A four-point Likert scale (very satisfied, satisfied, normal or dissatisfied) was utilized to record the outcomes. At 6 months, 1 year and 2 years postoperatively the HSS and SF-12 score were used to evaluate functional recovery and health related quality of life. HSS score is a scoring system for evaluating knee joint function which total score was 100. The main items were pain evaluation, function score, range of motion, muscle strength, flexion deformity, joint stability. SF-12 quality of life scale mainly evaluates physical and mental health, including a total of 12 questions [17]. The total score of physiological function, physiological function, body pain and general health is the total physiological score (Physical Component Summary, PCS), while the total score of energy, social function, emotional function and mental health is psychological score (Mental Component Summary, MCS).

### Statistical analysis

All continuous data were presented as the mean ± standard deviation (95%CI Upper – lower limit) and were analyzed by independent-sample Student’s t tests. All discontinuous data were presented as frequencies (percentages) and were analyzed by Pearson’s χ^2^ tests or Fisher’s exact tests. The Kruskal-Wallis H test was used to analyze ordinal rank data such as satisfaction rate and PON/V. All raw significance levels were set at α = 0. 05, and *P* < 0.05 indicated a significant difference. VAS rest scores were determined as the primary outcome parameter to calculate the sample size. The 2-point difference was determined to be the minimum clinically important difference (MCID) because the average acceptable VAS pain score difference following surgery was approximately 1 to 2 points based on previous studies [18–20]. Minimal effect size (δ) more than 1 point, with a Power of 0.9 and α < 0.05, at least 23 patients per group were required [21]. 30 participants were allocated in each group, in case of withdrawal during the study. All statistical analyses were calculated using SAS 9.4 (Statistical Analysis System). The sample size was determined using Jamovi 2.3 (The jamovi project, retrieved from https://www.jamovi.org). The charts were drawn using GraphPad Prism 8.0 (GraphPad Software).

## Results

### Patient demographics

A CONSORT-compliant flow chart of the procedure and participants is shown in Fig. 1. A total of 86 patients were assessed for eligibility. Twenty-six of those patients were excluded, 14 patients did not meet the inclusion criteria, among whom 2 patients had severe cardiac disease, 1 patient had renal dysfunction, 1 patient used opioid in half a year. 12 patients refused to participate. The baseline characteristics and preoperative demographics of the patients are shown in Table 1. There were no significant differences in terms of characteristics, preoperative pain, function, or duration of surgery in the two groups. No patients in any of the groups were excluded from the analysis.

### Primary outcomes

**Fig. 2:**
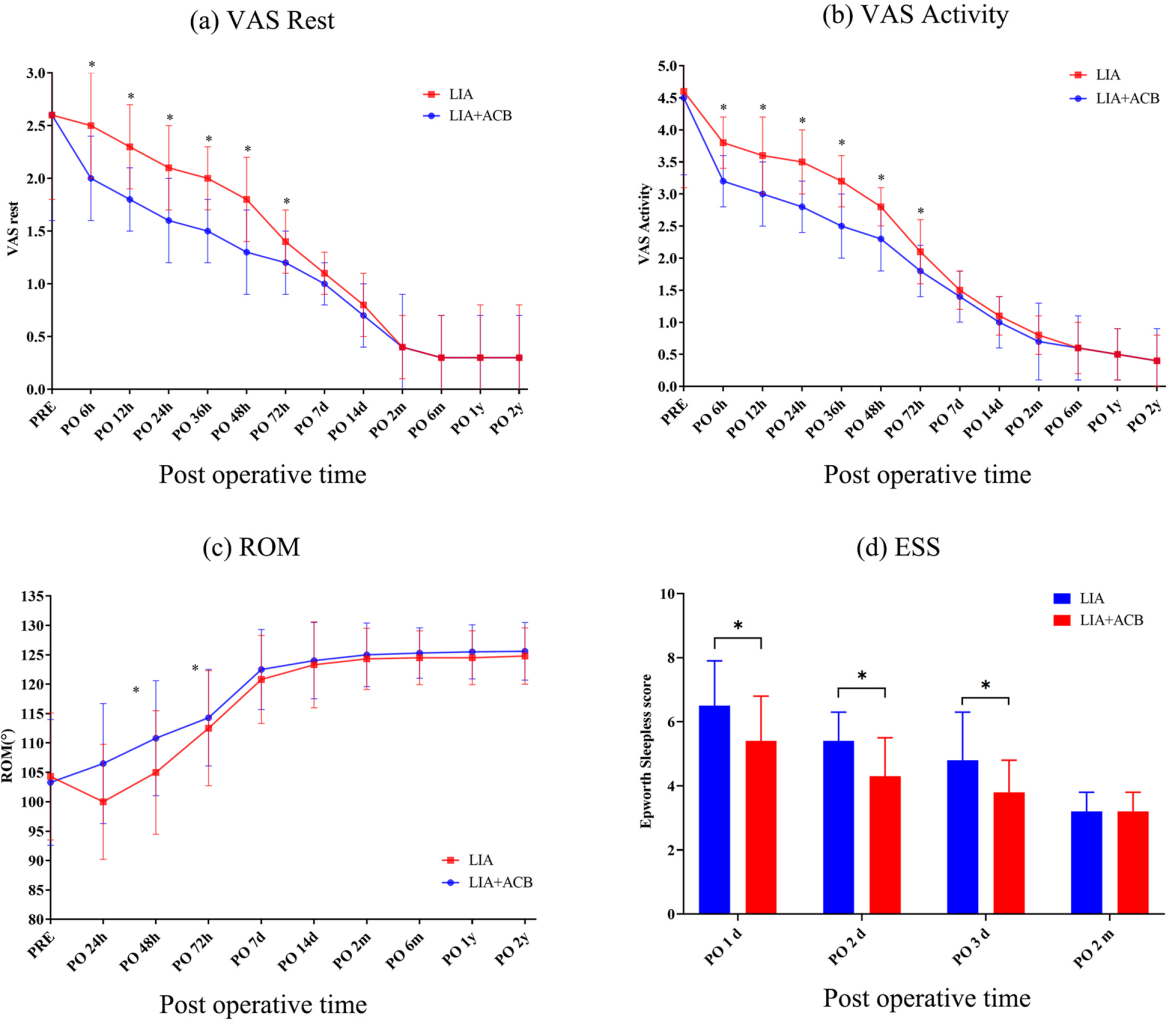
**a** Postoperative VAS resting pain scores, (**b**) postoperative VAS active pain scores, (**c**) postoperative range of motion, (**d**) postoperative Epworth sleepless scale. * *P* < 0.05, which indicate significate differences

The primary outcomes are listed in Table 2 and Fig. 2. For postoperative resting pain, all two groups had good pain control, with mean VAS scores lower than 3 (mild pain). In terms of rest or active pain, VAS rest pain was 2.1 ± 0.4 (1.95 to 2.24) in group A and 1.6 ± 0.4 (1.45 to 1.74) in group B at postoperative 24 hours. VAS active pain was 3.5 ± 0.5 (3.32 to 3.67) in group A and 3.5 ± 0.5 (3.32 to 3.67) in group B at postoperative 24 hours. Group A (LIA group) had significant more VAS pain than Group B (LIA + ACB group) up to postoperative 72 hours (*P* < 0.05), However, none of the between-group differences exceeds the MCID, No significant differences between two groups after PO 72 h to PO 2y (*P* > 0.05). Total postoperative opioid consumption was recorded at PO 24 h and 72 h. opioid consumption was 14.5 ± 5.2 mg in Group A, while only 8.2 ± 4.5 mg in Group B. LIA + ACB had significantly less opioid consumption than LIA. The location of pain was recorded at PO 48 h. LIA Group had more anterior knee pain than LIA + ACB group, while the posterior knee pain had no significant differences. Analgesic use from PO 14d to PO 2y did not significantly differ between the two groups. Regarding functional recovery, ROM showed significant differences between the two groups up to PO 48 h: the groups that received LIA + ACB had better ROM than LIA alone. There was no significant difference between the two groups after 48 h to 2y. Nearly all patients could complete the straight leg raise and get out of bed at PO 48 h, although there were significant differences up to PO 12 h, LIA + ACB had a better degree of completion in terms of straight leg raise. There were significant differences between the two groups in terms of the get out of bed test and the Walking 20 m test. The primary outcomes are listed in Table 2 and Fig. 2.

**Table 2:** Primary results

	Group A (*n* = 30)	Group B (*n* = 30)	t / χ^2^	P
**Pain**				
**VAS Rest**				
PO 6 h	2.5 ± 0.5 (2.32 to 2.67)	2.0 ± 0.4 (1.85 to 2.14)	4.277	0.0001* ^a^
PO 12 h	2.3 ± 0.4 (2.15 to 2.44)	1.8 ± 0.3 (1.69 to 1.90)	5.477	< 0.0001* ^a^
PO 24 h	2.1 ± 0.4 (1.95 to 2.24)	1.6 ± 0.4 (1.45 to 1.74)	4.841	< 0.0001* ^a^
PO 36 h	2.0 ± 0.3 (1.89 to 2.10)	1.5 ± 0.3 (1.39 to 1.60)	6.454	< 0.0001* ^a^
PO 48 h	1.8 ± 0.4 (1.65 to 1.94)	1.3 ± 0.4 (1.15 to 1.44)	4.841	< 0.0001* ^a^
PO 72 h	1.4 ± 0.3 (1.29 to 1.50)	1.2 ± 0.3 (1.09 to 1.30)	2.582	0.0124* ^a^
PO 7d	1.1 ± 0.3 (0.99 to 1.20)	1.0 ± 0.2 (0.92 to 1.07)	1.518	0.1342 ^a^
PO 14d	0.8 ± 0.3 (0.69 to 0.90)	0.7 ± 0.3 (0.59 to 0.80)	1.291	0.2018 ^a^
PO 2 m	0.4 ± 0.3 (0.29 to 0.50)	0.4 ± 0.5 (0.22 to 0.57)	–	1.0000 ^a^
PO 6 m	0.3 ± 0.4 (0.15 to 0.44)	0.3 ± 0.4 (0.15 to 0.44)	–	1.0000 ^a^
PO 1y	0.3 ± 0.5 (0.12 to 0.47)	0.3 ± 0.4 (0.15 to 0.44)	–	1.0000 ^a^
PO 2y	0.3 ± 0.5 (0.12 to 0.47)	0.3 ± 0.4 (0.15 to 0.44)	–	1.0000 ^a^
**VAS Activity**				
PO 6 h	3.8 ± 0.4 (3.65 to 3.94)	3.2 ± 0.4 (3.05 to 3.34)	5.801	< 0.0001* ^a^
PO 12 h	3.6 ± 0.6 (3.38 to 3.81)	3.0 ± 0.5 (2.82 to 3.17)	4.207	0.0001* ^a^
PO 24 h	3.5 ± 0.5 (3.32 to 3.67)	3.5 ± 0.5 (3.32 to 3.67)	5.988	< 0.0001* ^a^
PO 36 h	3.2 ± 0.4 (3.05 to 3.34)	2.5 ± 0.5 (2.32 to 2.67)	5.988	< 0.0001* ^a^
PO 48 h	2.8 ± 0.3 (2.69 to 2.90)	2.3 ± 0.5 (2.12 to 2.47)	4.696	< 0.0001* ^a^
PO 72 h	2.1 ± 0.5 (1392 to 2.27)	1.8 ± 0.4 (1.65 to 1.94)	2.566	0.129* ^a^
PO 7d	1.5 ± 0.3 (1.39 to 1.60)	1.4 ± 0.4 (1.25 to 1.54)	1.095	0.2778 ^a^
PO 14d	1.1 ± 0.3 (0.99 to 1.20)	1.0 ± 0.4 (0.85 to 1.14)	1.095	0.2778 ^a^
PO 2 m	0.8 ± 0.3 (0.69 to 0.90)	0.7 ± 0.3 (0.59 to 0.80)	1.291	0.2018 ^a^
PO 6 m	0.6 ± 0.4 (0.45 to 0.74)	0.6 ± 0.5 (0.42 to 0.77)	–	1.0000 ^a^
PO 1y	0.5 ± 0.4 (0.35 to 0.0.64)	0.5 ± 0.4 (0.35 to 0.64)	–	1.0000 ^a^
PO 2y	0.4 ± 0.4 (0.25 to 0.54)	0.4 ± 0.5 (0.22 to 0.57)	–	1.0000 ^a^
**Location of Pain PO 48 h**				
Anterior knee	21(70%)	12(40%)	5.454	0.0195* ^b^
Posterior pain	12(40%)	7(23.3%)	1.925	0.1652 ^b^
**Opioid consumption**				
PO 24 h (mg)	6.8 ± 4.3	3.6 ± 1.5	3.848	0.0003* ^a^
PO 72 h (mg)	14.5 ± 5.2	8.2 ± 4.5	5.017	< 0.0001* ^a^
**Analgesic used PO 14d to 1y**				
Acetaminophen	3 (10%)	2(6.7%)	–	1.0000 ^c^
COX-2	5 (16.7%)	3(10%)	–	0.7065 ^c^
Opioids	0 (0%)	0 (0%)	–	1.0000 ^c^
**Function**				
**ROM**				
PO 24 h	100.0 ± 9.8 (96.49 to 103.50)	106.5 ± 10.2 (102.85 to 110.15)	3.291	0.0146* ^a^
PO 48 h	105 ± 10.5 (101.24 to 108.75)	110.8 ± 9.8 (107.29 to 114.23)	2.211	0.0309* ^a^
PO 72 h	112.5 ± 8.9 (109.31 to 115.68)	114.3 ± 8.2 (120.06 to 124.93)	0.815	0.4186 ^a^
PO 7d	120.8 ± 7.5 (118.12 to 123.48)	122.5 ± 6.8 (121.67 to 126.32)	0.919	0.3615 ^a^
PO 14d	123.3 ± 7.3 (120.68 to 125.91)	124 ± 6.5 (123.06 to 126.93)	0.392	0.6963 ^a^
PO 2 m	124.3 ± 5.2 (122.43 to 126.16)	125 ± 5.4 (123.76 to 126.83)	0.511	0.6110 ^a^
PO 6 m	124.5 ± 4.6 (122.85 to 126.14)	125.3 ± 4.3 (123.06 to 126.93)	0.608	0.5450 ^a^
PO 1y	124.5 ± 4.6 (122.85 to 126.14)	125.5 ± 4.6 (123.85 to 127.14)	0.842	0.4030 ^a^
PO 2y	124.8 ± 4.8 (123.08 to 126.52)	125.6 ± 4.9 (123.84 to 127.35)	0.725	0.5255 ^a^
**Straight leg raise test**				
PO 24 h	19 (63.3%)	26 (93.3%)	4.356	0.0369* ^b^
PO 48 h	26 (93.3%)	29 (96.6%)	–	0.3533 ^c^
PO 72 h	30 (100%)	30 (100%)	–	1.0000 ^c^
**Get out of bed test**				
PO 24 h	23 (76.6%)	25 (83.3%)	0.416	0.5186 ^b^
PO 48 h	30 (100%)	30 (100%)	–	1.0000 ^c^
**Walking 20 m test**				
PO 24 h	18 (60%)	20 (66.7%)	0.287	0.5921 ^b^
PO 48 h	26 (93.3%)	29 (96.6%)	–	0.3533 ^c^
PO 72 h	30 (100%)	30 (100%)	–	1.0000 ^c^
**Epworth Sleepless score**				
PO 1st d	6.5 ± 1.4 (5.99 to 7.00)	5.4 ± 1.4 (4.89 to 5.90)	3.043	0.0035* ^a^
PO 2nd d	5.4 ± 0.9 (5.07 to 5.72)	4.3 ± 1.2 (3.87 to 4.72)	4.017	0.0002* ^a^
PO 3rd d	4.8 ± 1.5 (4.26 to 5.33)	3.8 ± 1.0 (3.44 to 4.15)	3.038	0.0036* ^a^
PO 2 m	3.2 ± 0.6 (2.98 to 3.41)	3.2 ± 0.5 (3.02 to 3.37)	–	1.0000 ^a^

*VAS* visual analogue scale, *ROM* range of motion, *PO* post-operative

^a^ The P value represented the result of the Student’s t test for continuous variables between 2 groups

^b^ The P value represented the result of the Pearson’s χ^2^ test for discontinues variables between 2 groups

^c^ The *P* value represented the result of the Fisher’s exact test for discontinues variables between 2 groups

*P* < 0.05 indicated significant differences

### Secondary outcomes

Regarding the length of hospitalization, no significant differences were found, as shown in Table 3. Regarding adverse events, 4 (13.3%) patients in Group A and 3 (10%) patients in Group B had mild PON/V. 2 (6.6%) patients in Group A and 1 (3.3%) patients in Group B had moderate PON/V. 2 (6.6%) in both Two groups had uroschesis, and catheters were installed only on the day after surgery. Only one (3.3%) patient had pruritus in Group A. The two groups had no significant differences in PON/V, uroschesis, or pruritus. No other adverse events, such as dizziness, hypotension or wound infection, were found, as shown in Table 3. the LIA + ACB group had significant better satisfaction rate of pain control than LIA alone as shown in Table 3, and the satisfaction rate of function recovery had no significant differences. HSS and SF-12 score had no no significant differences in 6 m to 2y follow-up, as shown in Table 3.

**Table 3:** Secondary results

	Group A (*n* = 30)	Group B (*n* = 30)	t/H	P
**Hospital stay (d)**	2.9 ± 0.5 (2.72 to 3.07)	2.7 ± 0.4 (2.55 to 2.84)	1.711	0.0925 ^a^
**Hospital Stay > 3d n (%)**	4 (13.3%)	3 (10%)	–	1.0000 ^c^
**HSS**				
PO 6 m	87.8 ± 4.8 (86.08 to 89.51)	88.6 ± 4.5 (86.98 to 90.21)	0.666	0.5081 ^a^
PO 1y	91.6 ± 4.0 (90.16 to 93.03)	91.8 ± 4.7 (90.11 to 93.48)	0.177	0.8597 ^a^
PO 2y	92.5 ± 5.6 (90.49 to 94.50)	92.7 ± 5.8 (90.62 to 94.77)	0.136	0.8924 ^a^
**SF-12(PCS)**				
PO 6 m	21.0 ± 3.9 (19.60 to 22.39)	21.4 ± 4.3 (19.86 to 22.93)	0.377	0.7073 ^a^
PO 1y	22.7 ± 4.1 (21.23 to 24.16)	22.8 ± 4.3 (21.26 to 24.33)	0.092	0.9269 ^a^
PO 2y	23.1 ± 5.8 (21.02 to 25.17)	23.3 ± 5.6 (21.29 to 25.30)	0.136	0.8924 ^a^
**SF-12(MCS)**				
PO 6 m	24.3 ± 2.9 (23.26 to 25.33)	24.5 ± 3.2 (23.35 to 25.64)	0.253	0.8007 ^a^
PO 1y	26.2 ± 2.7 (25.23 to 27.16)	26.4 ± 3.1 (25.29 to 27.50)	0.266	0.7908 ^a^
PO 2y	26.8 ± 3.0 (25.72 to 27.87)	26.9 ± 3.2 (25.75 to 28.04)	0.122	0.9026 ^a^
**Pain control**			5.468	**0.019**^**a**^
Very satisfied	15 (50%)	22 (73.3%)		
Satisfied	10 (33.3%)	6 (20%)		
Normal	5 (16.6%)	2 (6.6%)		
Dissatisfied	0 (0%)	0 (0%)		
**Function recovery**			1.586	0.208^**a**^
Very satisfied	17 (62.5%)	19 (63.3%)		
Satisfied	12 (40%)	11 (36.6%)		
Normal	1 (3.3%)	0 (0%)		
Dissatisfied	0 (0%)	0 (0%)		

*HSS* Hospital for special surgery score, *SF-12* 12 short form scale, *PCS* Physical Component Summary, *MCS* Mental Component Summary, *PO* post-operative

^a^ The P value represented the result of the Kruskal-Wallis H test for ranked data between 2 groups

*P* < 0.05 indicates significant differences

## Discussion

From the results of the study, important findings were observed that supported our hypotheses: ACB (ropivacaine) combined with LIA (cocktail of ropivacaine, morphine and betamethasone) provided significantly better postoperative analgesic effects and pain control than LIA alone; reduced opioid consumption up to PO 48 h; while the pain differences between two groups are below the MCID. Moreover, ACB combined with LIA enhanced early recovery in terms of functional measures such as ROM, leg raising; and improved sleep quality.

Multimodal analgesia has become the most important and effective method in the ERAS model for pain management as applied to TKA. Effective pain management is conducive to early rehabilitation and improves the patient satisfaction rate [15, 22]. LIA has been regarded as an alternative technique for pain control. LIA with ropivacaine alone may provide effective analgesic effects [23]. Moreover, ropivacaine combined with steroids may have better analgesic effects than ropivacaine alone. Ikeuchi et al. [8] evaluated 20 patients who received ropivacaine alone and 20 patients who received peri-articular injections of ropivacaine and dexamethasone; they found that pain severity was significantly lower in the steroid group than in the control group within 3 days postoperatively; thus, adding steroids to local infiltration analgesia resulted in significant early pain relief in TKA. Betamethasone is a long-acting glucocorticoid with potential anti-inflammatory and pain control properties. In our study, the addition of 10 mg morphine and 5 mg betamethasone to ropivacaine LIA significantly improved its analgesic effect and pain control up to PO 72 h. The chronic use of steroids may increase the risk of complications such as hyperglycemia, gastric ulcers, and wound infections. Morphine may cause complications such as nausea, pruritus, hypotension, delayed mobilization, and urinary retention [24, 25]. However, previous studies also did not demonstrate differences in serious adverse effects after periarticular steroid or morphine injection in TKA [1, 2, 8, 24, 25]. A meta-analysis revealed that small doses periarticular injection steroids did not increase complications after TKA [7]. Additionally, our study found no significant differences among the four groups in the incidence of complications. LIA with morphine and betamethasone did not increase the number of adverse events.

FNB is a kind of nerve block which has been widely used in total knee arthroplasty to reduce pain. However, FNB may reduce the strength of quadriceps muscles, so as to increase the risk of falls, and may be harmful to early rehabilitation [10, 15, 23, 26]. ACB has similar analgesic effect to FNB, while ACB has less effect on the strength of quadriceps muscles, increases walking stability of the patients, and is beneficial to recover function earlier [12]. Both LIA and ACB are useful method for multimodal analgesia management of TKA; However, the effect of combined application is still controversial. Grosso et al. [11] compared ACB combined with LIA to ACB alone or LIA alone; they demonstrated that ACB combined with LIA achieved significantly lower pain scores and opioid consumption than ACB alone over a 48 h postoperative period, but ACB with LIA showed no significant difference from to LIA alone with regard to either pain or opioid consumption. Li et al. [14] conducted a meta-analysis comparing ACB combined with LIA to LIA alone, demonstrating that combined ACB with LIA significantly reduced pain scores and morphine consumption compared to LIA alone. However, this meta-analysis included only randomized controlled trials with no more than 30 participants per group and had a maximum follow-up period of 2 weeks. Our study compared ACB with LIA to LIA alone and found that LIA recipients had less anterior knee pain. Due to a mathematical significant difference that may not be clinically significant. Minimal clinically important difference (MCID) defines the minimal clinical benefit of an intervention. The average acceptable MCID for the VAS pain score after TKA was approximately 1 to 2 points according to previous studies [18–20]. The 2-point difference was determined to be MCID in our study, however, no pain difference reached the MCID. This indicated that the pain have a small range of changes and does not completely reach a significant clinical difference. Although pain difference did exceed MCID, ACB combined LIA still have significantly less opioid consumption than LIA alone (*P* < 0.05). This result was similar to the findings of Nader et al. [13], who compared 20 recipients of ACB combined with LIA to 20 recipients of LIA alone, revealing that ACB with bupivacaine 0.25% and epinephrine 1:300,000 effectively reduced pain and the quantity of opioids required. ACB + LIA may be an effective form of pain management for patients undergoing TKA.

Our study had some shortcomings. First, the samples were rather small; thus, the pairwise differences between groups were often statistically nonsignificant. We often used conservative statistical methods for comparisons between groups; thus, an increased sample size might help reveal the significant differences. Second, our sample contained more females than males, and the recovery process may be different for different genders. Finally, in order to increase the sensitivity of the functional results, the accuracy of the tests may need to be improved.

## Conclusion

ACB combined with LIA provide better early pain control. Although the small statistical benefit may not result in minimal clinically important difference, ACB combined with LIA also reduce opioid requirements, improve sleeping quality, and do not increase the complication rate. Therefore, ACB combined with LIA still have good application prospects as an effective pain management for TKA, and it is suggested that the combined application should be used to better control knee pain.

## Data Availability

The datasets used and/or analysed during the current study are available from the corresponding author on reasonable request.

## Abbreviations

TKA: Total knee arthroplasty
LIA: Local infiltration analgesia
ACB: Adductor canal block
VAS: Visual analogue scale
ESS: Epworth sleepless scale
ROM: Range of motion
HSS: Hospital for special surgery score
SF-12: 12 short form scale
PCS: Physical Component Summary
MCS: Mental Component Summary
PO: Post-operative
